# Effects of public health interventions and zero COVID policy on paediatric diseases: A Southern China study

**DOI:** 10.7189/jogh.14.05011

**Published:** 2024-01-26

**Authors:** Li Huang, Chen Yang, Huoyun Pan, Yiling Gu, Ling Li, Meng Kou, Shaoxiang Chen, Jianlong Wu, Jiacee Lian, Jinqiu Zhang, Jiaowei Gu, Rui Wei, Hao Chen, Sitang Gong, Hongwei Zhang, Yi Xu, Qizhou Lian

**Affiliations:** 1 Prenatal Diagnostic Center and Cord Blood Bank, Guangzhou Women and Children's Medical Center, Guangzhou Medical University, Guangzhou, China; 2 Faculty of Synthetic Biology, Shenzhen Institute of Advanced Technology, Chinese Academy of Sciences, Shenzhen, China; 3 Department of Pharmacy, The First Affiliated Hospital of Shenzhen University, Shenzhen Second People’s Hospital, Shenzhen, China; 4 Department of Pediatrics, Guangzhou Women and Children's Medical Center, Guangzhou Medical University, Guangzhou, China; 5 Department of Infectious Diseases, Guangzhou Children's Hospital, Guangzhou, China; 6 Department of Pediatrics, Guangzhou Maternal and Child Health Hospital, Guangzhou, China; 7 Clinical Data Center, Guangzhou Women and Children's Medical Center, Guangzhou Medical University, Guangzhou, China; 8 School of Health Sciences, Ngee Ann Polytechnic, Singapore; 9 Department of Pediatrics, Affiliated Taihe Hospital of Hubei University of Medicine, Shiyan, China; 10 Department of Gastroenterology, Guangdong Provincial People’s Hospital (Guangdong Academy of Medical Sciences), Southern Medical University, Guangzhou, China; 11 Department of Navy Epidemiology, Faculty of Naval Medicine, Naval Medical University, Shanghai, China; 12 Department of Surgery, The University of Hong Kong Shenzhen Hospital, Shenzhen, China; 13 Department of Medicine and State Key Laboratory of Pharmaceutical Biotechnology, the University of Hong Kong, Special Administrative Region China

## Abstract

**Background:**

With the spread of the severe acute respiratory syndrome coronavirus 2 (SARS-CoV-2) pandemic in schools and communities, clinical evidence is needed to determine the impact of the pandemic and public health interventions under the zero coronavirus disease policy on the occurrence of common infectious diseases and non-infectious diseases among children.

**Methods:**

The current study was designed to analyse the occurrence of common infectious diseases before and after the pandemic outbreak in southern China. Data was obtained for 1 801 728 patients admitted into children’s hospitals in Guangzhou between January 2017 and July 2022. Regression analysis was performed for data analysis.

**Results:**

The annual occurrence of common paediatric infectious diseases remarkably decreased after the pandemic compared to the baseline before the pandemic and the monthly occurrence. Cases per month of common paediatric infectious diseases were significantly lower in five periods during the local outbreak when enhanced public health measures were in place. Cases of acute non-infectious diseases such as bone fractures were not reduced. Non-pharmaceutical interventions decreased annual and monthly cases of paediatric respiratory and intestinal infections during the coronavirus disease 2019 (COVID-19) pandemic, especially when enhanced public health interventions were in place.

**Conclusions:**

Our findings provide clinical evidence that public health interventions under the dynamic zero COVID policy in the past three years had significant impacts on the occurrence of common respiratory and intestinal infectious diseases among children and adolescents but little impact on reducing non-infectious diseases such as leukaemia and bone fracture.

The severe acute respiratory syndrome coronavirus 2 (SARS-CoV-2) epidemic has lasted almost three years since it was declared a public health emergency of international concern in 2020 [1]. Over 770 million confirmed coronavirus disease 2019 (COVID-19) cases and more than 6.9 million deaths have been reported globally [2]. With the majority of medical resources directed at the rapid increase in patients hospitalised with COVID-19 infection, critically ill patients, and those with long COVID, the SARS-CoV-2 pandemic also impacted the clinical outcomes of patients without COVID-19 [3]. The situation was aggravated by limited access to hospitals due to community lockdowns. In particular, this affected adult patients with ST-segment elevation myocardial infarction [4] who had reduced access to medical care [5] and delays in treatment [6]. On the other hand, the incidence of influenza and other notifiable infectious diseases was reduced in the general population while public health interventions were in place [7-10]. Nevertheless, for population in China mainland, the impact of public health interventions before and after the pandemic on the occurrence of common paediatric infectious diseases and non-infectious diseases is yet unknown. This multicentre, retrospective, observational study was designed to analyse the occurrence of paediatric respiratory and intestinal infectious diseases in southern China, as well as non-infectious conditions such as acute bone fracture and leukaemia before and after the pandemic to determine the impact of public health interventions on the disease portfolio in children and adolescents.

## METHODS

### Study design and approval

We conducted retrospective, observational study at Guangzhou Women and Children’s Medical Centre, Guangzhou Children’s Hospital, and Guangzhou Maternal and Child Health Hospital with centre ethical approval from the institutional review board (IRB) of Guangzhou Women and Children’s Medical Centre (ethical approval number 2022-246A01). Requirement for informed consent was waived by the IRB of Guangzhou Women and Children’s Medical Centre since data were exacted from the medical database, anonymised, and collected only for scientific research.

### Data sources and patient population

We retrieved clinical data from both inpatient and outpatient databases in the participating hospitals for the period 1 January 2017 to 31 July 2022 and included demographic data as well as annual and monthly cases of paediatric diseases identified by the inclusion criteria. Medical data were included in the current study for patients who fulfilled the following criteria:

1. Diagnosed with acute upper respiratory tract infection (AURTI), influenza, scarlatina, acute bronchitis, bronchopneumonia, measles, hand, foot, and mouth disease (HFMD), varicella, infectious mononucleosis, rotavirus infection, adenovirus infection, diarrhoea, leukaemia or fracture,
2. Aged between 0–18 years.

Two researchers cross-checked the data for quality control and analysis was supervised by a specialist in epidemiological and hygiene statistics. The current study is reported based on a STROBE checklist (Table S1 in the **Online Supplementary Document**).

### Data analysis plan and definition

**Figure 1:**
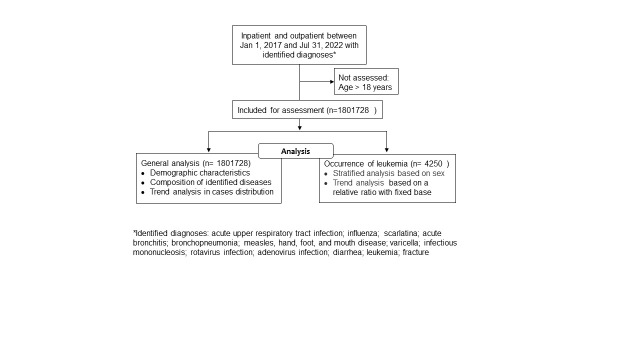
Data handling process and analysis diagram.

We collected annual and monthly cases of common paediatric infectious diseases, as well as cases of leukaemia and fracture. Occurrence of the latter remained relatively stable and served as a control group to detect the impact of the lockdown strategy on hospitalisation rates during the pandemic. Diseases identified by the inclusion criteria were expressed as a percentage of all cases of disease in the paediatric population. We calculated the average number of monthly cases of identified diseases each month from 1 January 2017 to 31 December 2019 to provide a baseline pre-pandemic. We compared the results with the same data retrieved after the pandemic. In addition, we analysed the impact of enhanced public health interventions including regional knockdown on the occurrence of identified diseases, as well as the occurrence of leukaemia in children and adolescents. Public health interventions were in accordance with the prevention and control strategy for COVID-19 issued by the National Health Commission of the People’s Republic of China [11] and related to mainly hand hygiene, mask wearing, physical distancing, improved ventilation, and vaccination for COVID-19 [12,13]. Enhanced public health measures also included timely disinfection, travel restrictions, school closures, reginal lockdown and local quarantine strategies [11]. Institutional review board approved the protocol prior to data collection (**Online Supplementary Document**). The data handling process and analysis are presented in **Figure 1**.

### Statistical analysis

We employed descriptive statistics to analyse the distribution features of variables. We used Shapiro-Wilk test for continuous variables to test their distribution features when the sample size was no more than 50, while Kolmogorov-Smirnov was used for variables with sample size less than 50. Categorical variables are presented as number and frequency. We performed the regression analysis using Joinpoint, version 4.8.0.1 (Bethesda, Maryland, USA) with annual percent change as a key index to evaluate the trends of annual occurrence of leukaemia from 2017–21. Statistical significance was set at *P*-value <0.05. No imputation was used for missing data.

## RESULTS

### Demographic features of patients and identified diseases

Study included 1 801 728 patients from January 2017 to July 2022 spanned all ages from childhood to adolescence but with a majority aged from zero to six years old (Figure S1 in the **Online Supplementary Document**), and 58.1% of patients were male (**Table 1**). Among these paediatric cases, the top three respiratory infectious diseases were AURTI (52.9%), acute bronchitis (16.8%), and bronchopneumonia (8.4%). Measles (4.9%) was a common notifiable infectious disease for paediatric patients, as was HFMD (3.7%), varicella, influenza, and scarlatina (**Table 2**). Intestinal infectious diseases included diarrhoea (9.5%) and rotavirus infection (0.4%). The number of cases of all diseases per year reduced remarkably after 2020 (Figure S2 in the **Online Supplementary Document**). The average cases per year of several diseases were significantly decreased over 50% (Table S2 in the **Online Supplementary Document**), including influenza (79.9%), HFMD (73.8%), scarlatina (60.7%), adenovirus infection (59.9%), rotavirus infection (59.2%), bronchopneumonia (57.1%), and varicella (53.6%).

**Table 1:** Demographic features of paediatric patients*

Demographic characteristics	2017	2018	2019	2020	2021	2022†	Total
Age in years							
*0–3*	227 581 (64.6)	246 717 (60.5)	236 440 (57.7)	130 747 (55.7)	135 142 (52.3)	60 575 (43.8)	1 037 202 (57.6)
*4–6*	98 589 (28.0)	126 911 (31.1)	130 706 (31.8)	81 308 (34.7)	98 955 (38.3)	58 476 (42.3)	594 945 (33.0)
*7–12*	23 484 (6.7)	30 471 (7.5)	38 140 (9.3)	19 412 (8.3)	21 112 (8.1)	16 765 (12.1)	149 384 (8.3)
*13–18*	2612 (0.7)	3528 (0.9)	5104 (1.2)	3126 (1.3)	3376 (1.3)	2451 (1.8)	20 197 (1.1)
Sex							
*Male*	207 932 (59.0)	238 299 (58.5)	237 282 (57.8)	134 479 (57.3)	149 380 (57.8)	79 414 (57.4)	1 046 786 (58.1)
*Female*	144 334 (41.0)	169 328 (41.5)	173 108 (42.2)	100 114 (42.7)	109 205 (42.2)	58 853 (42.6)	754 942 (41.9)

*Presented as n (%) unless specified otherwise.

†Data cut-off date was 31 July 2022.

**Table 2:** Paediatric patients with different diseases from January 2017 to July 2022, presented as n (%)

Diseases	2017	2018	2019	2020	2021	2022*	Total
AURTI	179 089 (50.8)	214 236 (52.6)	208 806 (50.9)	135 623 (57.8)	135 514 (52.4)	80 612 (58.3)	953 880 (52.9)
AB	49 148 (14.0)	69 959 (17.2)	70 200 (17.1)	38 219 (16.3)	49 884 (19.3)	24 783 (17.9)	302 193 (16.8)
BP	31 215 (8.9)	34 104 (8.4)	45 004 (11.0)	15 370 (6.5)	16 188 (6.3)	9055 (6.6)	150 936 (8.4)
IM	1210 (0.3)	1263 (0.3)	1361 (0.3)	1043 (0.4)	1290 (0.5)	501 (0.4)	6668 (0.4)
AI	1674 (0.5)	2117 (0.5)	4248 (1.0)	598 (0.3)	1551 (0.6)	657 (0.5)	10 845 (0.6)
Measles	15 650 (4.4)	19 190 (4.7)	17 556 (4.3)	13 675 (5.8)	15 323 (5.9)	7246 (5.2)	88 640 (4.9)
HFMD	22 921 (6.5)	14 714 (3.6)	18 834 (4.6)	2571 (1.1)	7304 (2.8)	1057 (0.8)	67 401 (3.7)
Varicella	2268 (6.0)	2441 (0.6)	1933 (0.5)	1047 (0.4)	1007 (0.4)	366 (0.3)	9062 (0.5)
Influenza	1261 (0.3)	2664 (0.6)	5836 (1.4)	1099 (0.5)	211 (0.1)	1299 (0.9)	12 370 (0.7)
Scarlatina	546 (0.2)	756 (0.2)	409 (0.1)	170 (0.1)	278 (0.1)	195 (0.1)	2354 (0.1)
Diarrhoea	41 824 (11.9)	40 780 (10.0)	31 639 (7.7)	21 358 (9.1)	25 308 (9.8)	9917 (7.2)	170 826 (9.5)
RI	2350 (0.7)	2135 (0.5)	1265 (0.3)	458 (0.2)	1105 (0.4)	324 (0.2)	7637 (0.4)
Fracture	2287 (0.7)	2430 (0.6)	2442 (0.6)	2501 (1.1)	2751 (1.1)	1600 (1.1)	14 011 (0.8)
Leukaemia	823 (0.2)	838 (0.2)	857 (0.2)	861 (0.4)	871 (0.3)	655 (0.5)	4905 (0.3)

AB – acute bronchitis, AI – adenovirus infection, AURTI – acute upper respiratory tract infection, BP – bronchopneumonia, HFMD – hand, foot, and mouth disease, IM – infectious mononucleosis, RI – rotavirus infection

*Data cut-off date was 31 July 2022.

### Distribution of diseases before and during the pandemic

**Figure 2:**
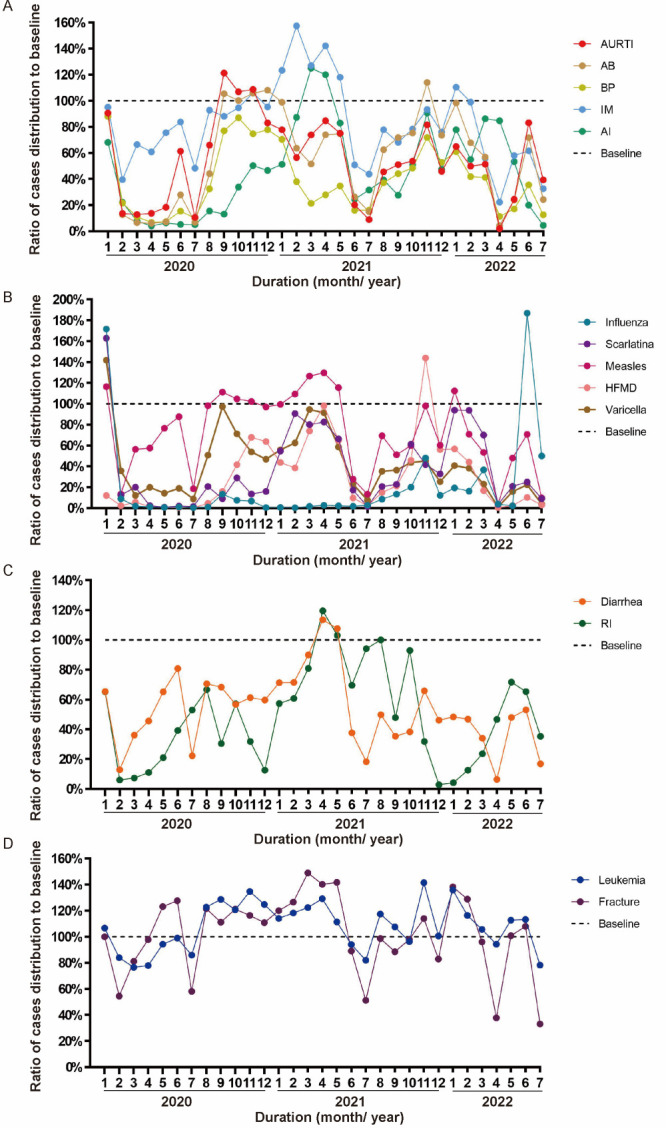
The ratio of changes in case distribution before and after the COVID-19 pandemic. **Panel A**. Common paediatric respiratory infectious diseases. **Panel B**. Notifiable infectious diseases. **Panel C**. Intestinal infectious diseases. **Panel D**. Monthly cases of leukaemia and fracture. AB – acute bronchitis, AI – adenovirus infection, AURTI – acute upper respiratory tract infection, BP – bronchopneumonia, HFMD – hand, foot, and mouth disease, IM – infectious mononucleosis, RI – rotavirus infection.

We calculated the monthly occurrence of identified diseases for years 2017–19, prior to the COVID-19 pandemic (Figure S3 in the **Online Supplementary Document**). The trend of monthly average cases over these three years were in line with the individual annual trends. Therefore, the monthly average cases of identified diseases could be considered a pre-pandemic baseline and was defined as 100% in order to calculate the ratio changes for each disease since 2020, post pandemic (**Figure 2**). Pre- and post-pandemic, the average number of cases per month of leukaemia and fracture was relatively stable while there was a downward trend post-2020 for monthly cases of common paediatric respiratory infectious diseases, notifiable infectious diseases, and intestinal infectious diseases (**Figure 2**, Panels A–D). In particular, remarkable decreases were noticed (Table S2 in the **Online Supplementary Document**) in HFMD (79.3%), influenza (63.8%), rotavirus infection (63.1%), varicella (60.0%), adenovirus infection (59.2%), bronchopneumonia (57.3%), and scarlatina (54.2%). A breakout peak was noticed for influenza at June 2022, which was out of the high-occurrence seasons of spring and winter. Further analysis for the cause of this outbreak was not performed due to resource limitation (**Figure 2****,** Panel B).

### The impact of enhanced public health measures on case distribution during the pandemic

**Figure 3:**
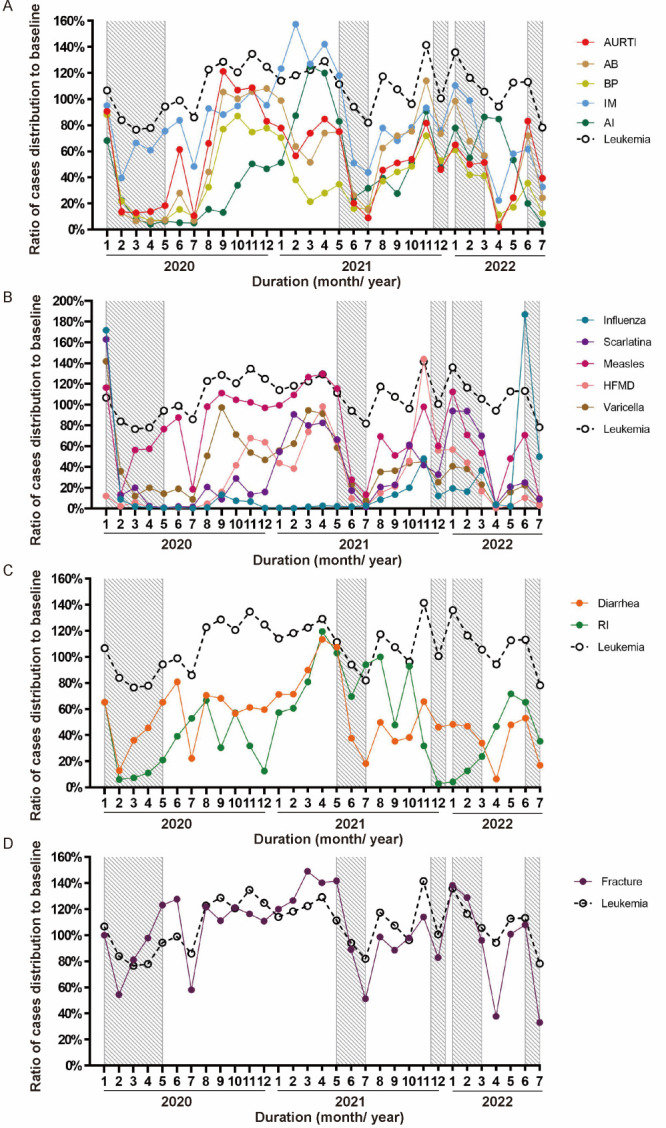
The impact of enhanced public health measures on the ratio of changes in case distribution during the pandemic. **Panel A**. Common paediatric respiratory infectious diseases. **Panel B**. Notifiable infectious diseases. **Panel C**. Intestinal infectious diseases. **Panel D**. as well as fracture. AB – acute bronchitis, AI – adenovirus infection, AURTI – acute upper respiratory tract infection, BP – bronchopneumonia, HFMD – hand, foot, and mouth disease, IM – infectious mononucleosis, RI – rotavirus infection.

We identified five periods of local outbreak in Guangzhou based on the confirmed cases of COVID-19 (Figure S4 in the **Online Supplementary Document**) and during periods when enhanced public health measures were implemented according to the dynamic zero-COVID policy. The number of cases per month of leukaemia during the pandemic was in line with that before the outbreak and served as a control to evaluate the impact of enhanced public health measures (**Figure 3**). Monthly cases of leukaemia remained relatively stable during the pandemic although a slight downward trend was noticed at the beginning of local outbreaks. During local outbreaks, downward trends were observed in the occurrence of all common paediatric infectious diseases identified in the current study with a decrease over 50% (**Figure 3**, Panels A–C). It was also noticed that the occurrence of influenza during the unexpected outbreak in May 2022, dropped dramatically with enhanced public health management (**Figure 3**, Panel B).

### The occurrence of leukaemia and fractures in children during the pandemic

We tested the distribution feature of leukaemia cases to be a normal distribution by Shapiro-Wilk analysis (*P* = 0.703). There was a slight upward trend in cases of leukaemia after 2017 with an annual percentage change of 1.466% (Figure S5 in the **Online Supplementary Document** and **Table 3**). Although a link with the pandemic, even with vaccination, could not be confirmed, a low annual percent change indicated a little impact of the pandemic and vaccination on the occurrence of leukaemia. In addition, an upward trend in monthly cases of fracture during the pandemic was suggested since case numbers were lower in only nine of 31 months compared with pre-pandemic baseline. We concluded that enhanced public health interventions, especially regional lockdowns and local quarantine strategies, did not impact occurrence of non-infectious diseases including leukaemia and bone fractures which require critical medical care during the pandemic.

**Table 3:** The occurrence of leukaemia in children from 2017 to 2021

Cases of leukaemia (n)	2017	2018	2019	2020	2021
Male	496	496	502	516	532
Female	327	342	355	345	338
Total, n (ratio)*	823 (1.00)	838 (1.02)	857 (1.04)	861 (1.05)	871 (1.06)

*The ratio was calculated as a relative ratio with a fixed base. The fixed base was set as the total case of leukaemia in 2017.

## DISCUSSION

We focused on the occurrence of paediatric respiratory and intestinal infectious diseases in Southern China before and after the pandemic to determine the effect of public health interventions on the disease portfolio of children and adolescents. We previously identified the clinical characteristics [14] and possibility of faecal-oral transmission of SARS-CoV-2 infection [15] in paediatric patients. Long term follow-up has been suggested to monitor children’s respiratory system development [16] based on the detection of SARS-CoV-2 infection in lung progenitor cells of children [17,18]. As well as the rapid spread of SARS-CoV-2 variants, the impact of the pandemic and enhanced public health measures on the occurrence and outcome of diseases other than COVID-19 has attracted the attention of health authorities. In China, a dynamic zero COVID-19 strategy was adopted in line with the prevention and control strategy of the health authority of China [11]. Public health measures were consequently stricter and more often imposed compared with other countries in Europe and America. Therefore, understanding the impact of the pandemic and enhanced public health measures on the occurrence of diseases other than COVID-19 would help determine the success of the COVID-19 control strategy in China.

Previous studies noted a decline in the incidence of infectious disease in populations across all ages, which was considered relative to non-pharmaceutical interventions [10,13,19]. A significant decline was reported in several infectious diseases including influenza [9,10,20,21], respiratory infections [22], and notifiable infectious diseases [7]. The overall frequency of community-acquired paediatric infections was reduced during strict application of non-pharmaceutical interventions in France [23]. Hospitalisations for seasonal respiratory virus infections also decreased in infants aged up to two years in New Zealand [24]. A reduce of respiratory tract infection-related visits was reported at paediatric emergency department in Taiwan [25]. Nonetheless, the impact on the occurrence of common paediatric infectious diseases as well as non-infectious conditions such as acute bone fracture and leukaemia was unknown in China mainland. In our current study of children mainly aged under six years, the annual occurrence of common paediatric infectious diseases declined remarkably and was in accordance with previous studies of a different population (Figure S1–2 in the **Online Supplementary Document**). Furthermore, monthly cases of common paediatric respiratory and intestinal infectious diseases demonstrated a downward trend after the pandemic compared with baseline while monthly cases of leukaemia and fracture showed only minor fluctuations (**Figure 2**). This downward trend in cases of common paediatric infectious diseases could not be explained by limited access to hospital care during the pandemic since number of cases hospitalised for leukaemia or fracture remained largely unchanged. Similarly, a study of care for patients with ST-segment elevation myocardial infarction care also suggested that restructuring of health services during the pandemic did not significantly adversely influence in-hospital outcomes [26]. In our study, further analysis revealed that the monthly number of patients with common paediatric infectious diseases was remarkably lower during five periods of local outbreak with enhanced public health measures (**Figure 3**). This suggests that non-pharmaceutical interventions may have played a role, consistent with previous studies [10,13,19]. Our data indicates that non-pharmaceutical interventions were important measures that could reduce the occurrence of respiratory and intestinal infectious diseases, particularly in children.

Recently, some concerns were raised about the safety of vaccines and the occurrence of leukaemia, although vaccination was recommended by local health authorities as an important strategy to prevent and decrease the number of severe cases of COVID-19 in those aged over 3 years [11]. In the current study, we determined that the pandemic and vaccination against COVID-19 had a low impact on the occurrence of leukaemia. Although further study with larger sample size is needed to draw a more confident conclusion, the risk of vaccines and tumorigenesis appears quite low. In addition the American Society of Clinical Oncology [27], American Cancer Society (ACS) [28], and National Comprehensive Cancer Network all recommend that patients with cancer be fully vaccinated against COVID-19 [29]. Besides, the safety of vaccination was also concerned in patients with autoimmune diseases. Increasing cases of new-onset autoimmune disorders after COVID-19 vaccination had been reported [30]. Moreover, it was indicated that SARS-CoV-2 infection was a trigger to autoimmune diseases [31], and COVID-19 was associated with a different degree of risk for various autoimmune diseases [32,33]. Preliminary analysis was also performed in paediatric patients, although the sample size was limited [34,35]. However, the impact of the pandemic and public health interventions on autoimmune diseases, especially for vaccination, was not analysed in the current study since we focus on common infectious diseases based on the resources of investigational centres, which could be explored in further studies.

### Study limitations

The study was conducted in three hospitals in the same city in Southern China. Therefore, the generalisability of our findings is limited without representative data from other regions in China, like Northern China. Second, the sample size for the analysis of the impact of vaccination on leukaemia occurrence was limited. The occurrence of paediatric infectious diseases in community could not be analysed since our population was limited in hospitalized paediatric patients based on the resources of investigational centres, while community health care was also important for public health management in the pandemic.

### Further study

Along with the declaration of World Health Organization (WHO) to end the COVID-19 emergency in May 2023, the focus of pandemic management was turned from emergency response to long-term COVID-19 disease management [36]. Based on the WHO guidance of calibrating the response, COVID-19 vaccination was suggested integrating into life course vaccination programs [37], and reach an aspirational target of 100% vaccination coverage among those in the highest priority groups [38]. Therefore, further study is required to improve vaccines that reduce transmission and have broad applicability [37], as well as the observation and monitoring about the safety of vaccination in different populations under various conditions, like autoimmune diseases. According to the WHO statement, further studies are also supported to understand the full spectrum, incidence and impact of post COVID-19 condition and the evolution of SARS-COV-2 in immunocompromised populations [37]. Based on the implementation of our study, a collaboration of institutions in different regions, including Community Healthcare Centre and Centres for Disease Control and Prevention, is suggested to improve the generalizability of data analysis with larger samples.

## CONCLUSIONS

The current study analysed the trends of common paediatric respiratory and intestinal infectious diseases before and after the pandemic. Non-pharmaceutical interventions were associated with a decrease in annual and monthly cases during the pandemic, especially during periods of local outbreaks when enhanced public health measures were in place. Notwithstanding the relatively limited sample size for the analysis of leukaemia occurrence, vaccination appeared to have a low impact. Our study provides clinical evidence that non-pharmaceutical interventions impact the occurrence of COVID-19 and that of common respiratory and intestinal infectious diseases in children and adolescents. However, little impact of COVID-19 pandemic and enhanced public health measures on reducing non-infectious diseases such as leukaemia and bone fracture in children.

## Additional material


Online Supplementary Document
