# Supplementary material for: Effects of public health interventions and zero COVID policy on paediatric diseases: A Southern China study
Source: J Glob Health. 2024 Jan 26;14:05011. doi: 10.7189/jogh.14.05011 (PMC10811438; doi:10.7189/jogh.14.05011)
Supplement: Online Supplementary Document [file jogh-14-05011-s001.pdf]

Effects of Public Health Interventions and Zero-COVID Policy on Pediatric Diseases: A Southern China Study

Online Supplementary Document

Table S1 STROBE checklist.

Table S2 The changes in average annual and monthly cases of common pediatric diseases before and after the COVID-19 pandemic.

Figure S1 The age distribution of included patients.

Figure S2 Disease distribution during the pandemic.

Figure S3 The case distribution of the identified pediatric diseases pre-COVID-19 pandemic (from 2017 to 2019).

Figure S4 The distribution of confirmed cases of COVID-19 in Guangzhou.

Figure S5 The case distribution for pediatric patients with leukemia.

Text S1 Study protocol.

Table S1 STROBE checklist.

	Item No	Recommendation	Page No
**Title and abstract**	1	(*a*) Indicate the study's design with a commonly used term in the title or the abstract	1
		(*b*) Provide in the abstract an informative and balanced summary of what was done and what was found	3
**Introduction**			
Background/rationale	2	Explain the scientific background and rationale for the investigation being reported	5
Objectives	3	State specific objectives, including any prespecified hypotheses	5
**Methods**			
Study design	4	Present key elements of study design early in the paper	5
Setting	5	Describe the setting, locations, and relevant dates, including periods of recruitment, exposure, follow-up, and data collection	6
Participants	6	(*a*) *Cohort study*—Give the eligibility criteria, and the sources and methods of selection of participants. Describe methods of follow-up<br>*Case-control study*—Give the eligibility criteria, and the sources and methods of case ascertainment and control selection. Give the rationale for the choice of cases and controls<br>*Cross-sectional study*—Give the eligibility criteria, and the sources and methods of selection of participants	6
		(*b*) *Cohort study*—For matched studies, give matching criteria and number of exposed and unexposed<br>*Case-control study*—For matched studies, give matching criteria and the number of controls per case	NA
Variables	7	Clearly define all outcomes, exposures, predictors, potential confounders, and effect modifiers. Give diagnostic criteria, if applicable	6
Data sources/ measurement	8*	For each variable of interest, give sources of data and details of methods of assessment (measurement). Describe comparability of assessment methods if there is more than one group	6
Bias	9	Describe any efforts to address potential sources of bias	6
Study size	10	Explain how the study size was arrived at	Figure1
Quantitative variables	11	Explain how quantitative variables were handled in the analyses. If applicable, describe which groupings were chosen and why	6-7
Statistical methods	12	(*a*) Describe all statistical methods, including those used to control for confounding	7
		(*b*) Describe any methods used to examine subgroups and interactions	7
		(*c*) Explain how missing data were addressed	7
		(*d*) *Cohort study*—If applicable, explain how loss to follow-up was addressed<br>*Case-control study*—If applicable, explain how matching of cases and controls was addressed<br>*Cross-sectional study*—If applicable, describe analytical methods taking account of sampling strategy	NA
		(*e*) Describe any sensitivity analyses	NA

Results

Participants	13*	(a) Report numbers of individuals at each stage of study—eg numbers potentially eligible, examined for eligibility, confirmed eligible, included in the study, completing follow-up, and analysed	Figure 1
		(b) Give reasons for non-participation at each stage	Figure 1
		(c) Consider use of a flow diagram	Figure 1
Descriptive data	14*	(a) Give characteristics of study participants (eg demographic, clinical, social) and information on exposures and potential confounders	8

		(b) Indicate number of participants with missing data for each variable of interest	Figure 1
		(c) *Cohort study*—Summarise follow-up time (eg, average and total amount)	NA
Outcome data	15*	*Cohort study*—Report numbers of outcome events or summary measures over time	8-9
		Case-control study—Report numbers in each exposure category, or summary measures of exposure	NA
		Cross-sectional study—Report numbers of outcome events or summary measures	NA
Main results	16	(*a*) Give unadjusted estimates and, if applicable, confounder-adjusted estimates and their precision (eg, 95% confidence interval). Make clear which confounders were adjusted for and why they were included	8
		(*b*) Report category boundaries when continuous variables were categorized	NA
		(*c*) If relevant, consider translating estimates of relative risk into absolute risk for a meaningful time period	NA
Other analyses	17	Report other analyses done—eg analyses of subgroups and interactions, and sensitivity analyses	9
**Discussion**			
Key results	18	Summarise key results with reference to study objectives	10
Limitations	19	Discuss limitations of the study, taking into account sources of potential bias or imprecision. Discuss both direction and magnitude of any potential bias	12
Interpretation	20	Give a cautious overall interpretation of results considering objectives, limitations, multiplicity of analyses, results from similar studies, and other relevant evidence	10-12
Generalisability	21	Discuss the generalisability (external validity) of the study results	12
**Other information**			
Funding	22	Give the source of funding and the role of the funders for the present study and, if applicable, for the original study on which the present article is based	3, 13

*Give information separately for cases and controls in case-control studies and, if applicable, for exposed and unexposed groups in cohort and cross-sectional studies.

Note: An Explanation and Elaboration article discusses each checklist item and gives methodological background and published examples of transparent reporting. The STROBE checklist is best used in conjunction with this article. Information on the STROBE Initiative is available at www.strobe-statement.org.

Abbreviations: NA, not applicable.

Table S2 The changes in average annual and monthly cases of common pediatric diseases before and after the COVID-19 pandemic.

Diseases	Average annual cases				Average monthly cases			
	2017-2019	2020-2021	Changes	%	2017-2019	2020-2022[b]	Changes	%
AURTI	200710	135569	-65141	-32.5	16726	11370	-5356	-32.0
AB	63102	44052	-19050	-30.2	5259	3627	-1632	-31.0
BP	36774	15779	-20995	-57.1	3065	1308	-1757	-57.3
IM	1278	1167	-111	-8.7	107	89	-18	-16.8
AI	2680	1075	-1605	-59.9	223	91	-132	-59.2
Measles	17465	14499	-2966	-17.0	1455	1151	-304	-20.9
HFMD	18823	4938	-13885	-73.8	1569	325	-1244	-79.3
Varicella	2214	1027	-1187	-53.6	185	74	-111	-60.0
Influenza	3254	655	-2599	-79.9	271	98	-173	-63.8
Scarlatina	570	224	-346	-60.7	48	22	-26	-54.2
Diarrhea	38081	23333	-14748	-38.7	3173	1769	-1404	-44.2
RI	1917	782	-1135	-59.2	160	59	-101	-63.1
Fracture	2386	2626	240	10.1	199	222	23	11.6
Leukemia	839	866	27	3.2	70	79	9	12.9

[a] Changes were calculated as the difference of the cases after the pandemic compared with the baseline before the pandemic.

[b] Data cut-off date was 31 July, 2022.

Abbreviations: AB, acute bronchitis; AI, adenovirus infection; AURTI, acute upper respiratory tract infection; BP, bronchopneumonia; HFMD, hand, foot, and mouth disease; IM, infectious mononucleosis; RI, rotavirus infection

Figure S1 The age distribution for included patients.

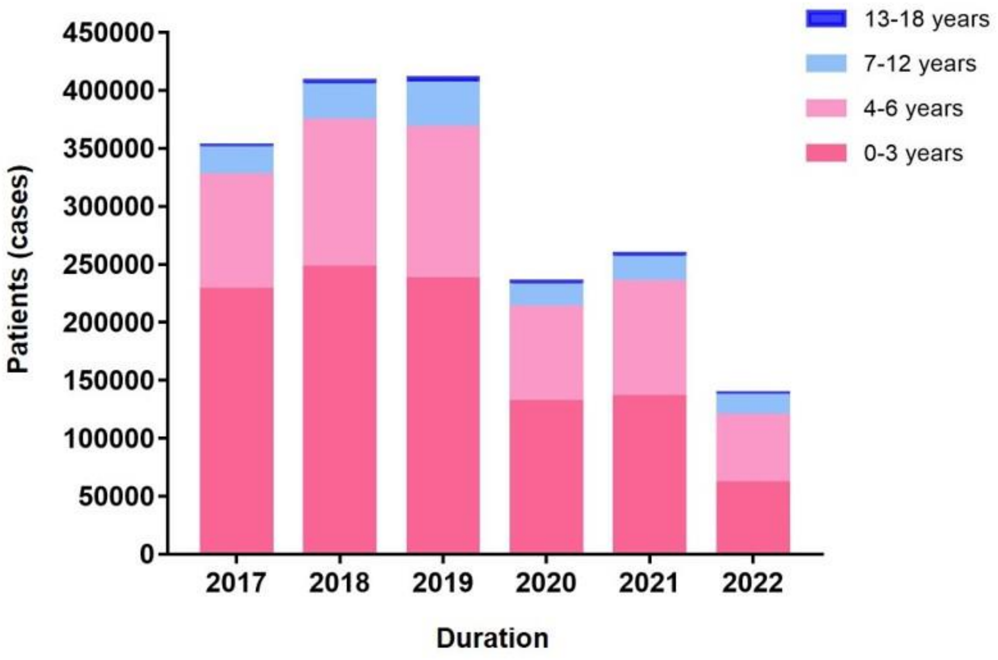

Figure S2 The case distribution of identified diseases during the pandemic.

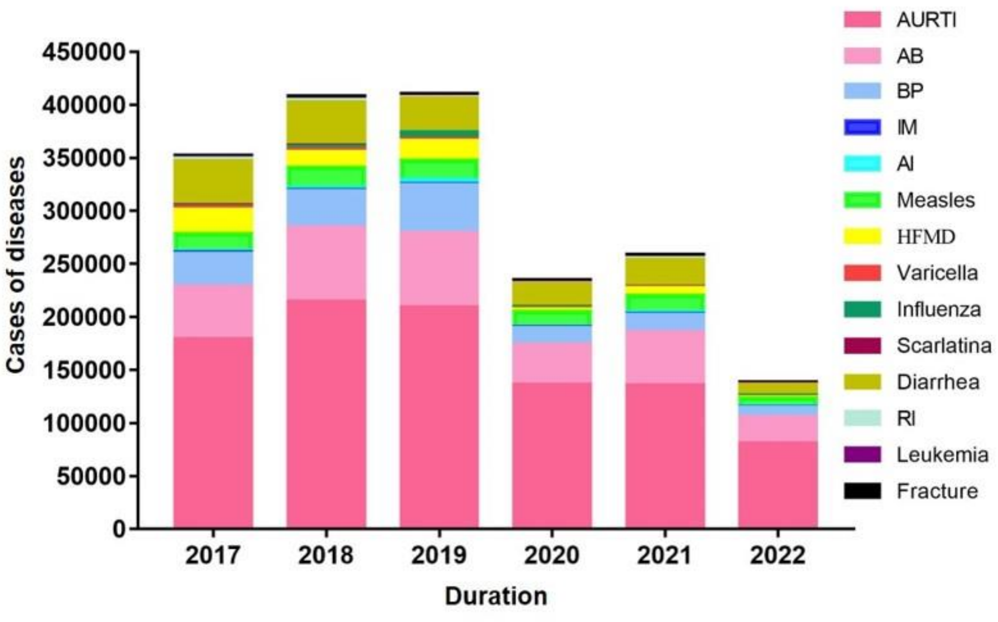

Figure S3 The case distribution of the identified pediatric diseases pre-COVID-19 pandemic (from 2017 to 2019).

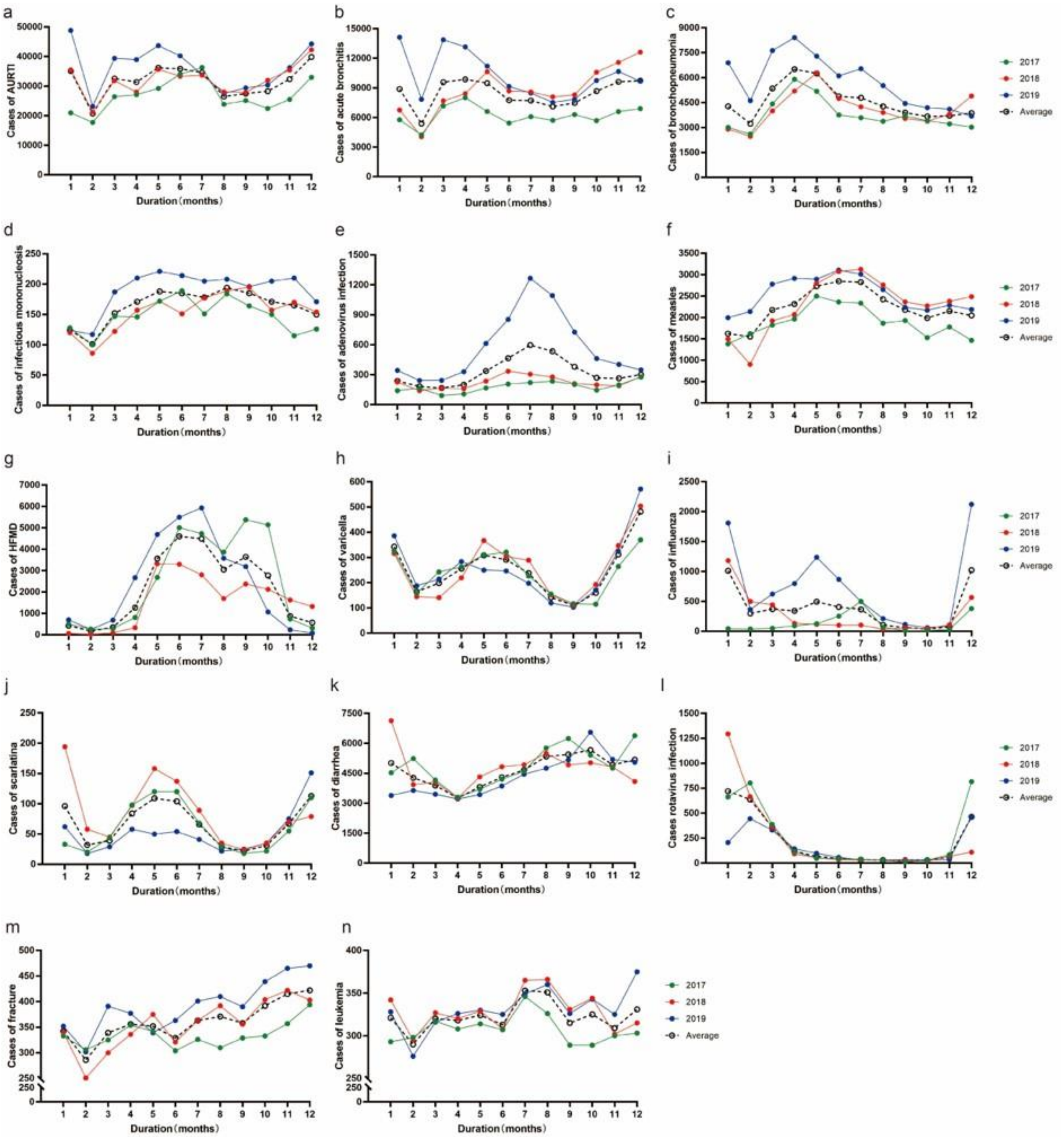

Figure S4 The distribution of confirmed cases of COVID-19 in Guangzhou.

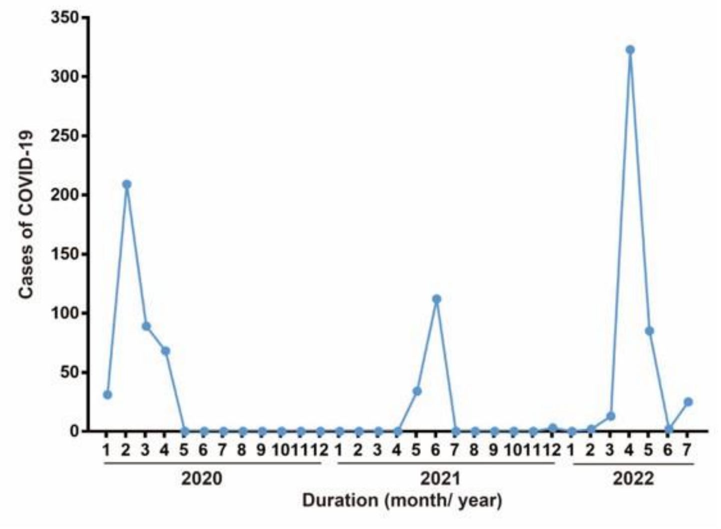

Figure S5 The case distribution for pediatric patients with leukemia.

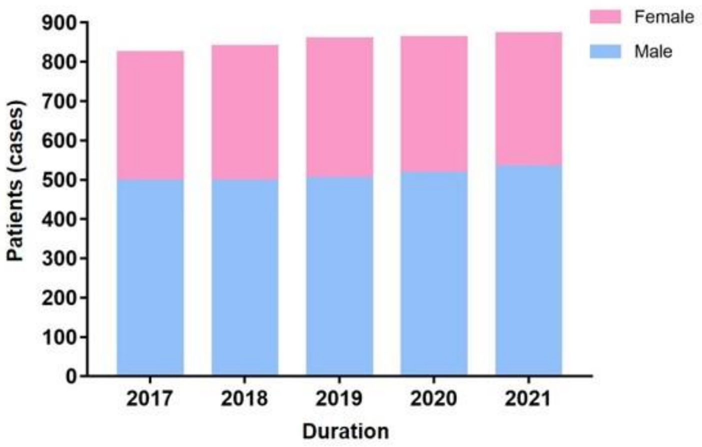

Text S1 Study protocol.

Effects of Public Health Interventions and Zero-COVID Policy on Pediatric Diseases: A Southern China Study

Version number: 1.0

Version date: 8 May 2022

1. Background and rationale

The severe acute respiratory syndrome coronavirus 2 (SARS-CoV-2) epidemic has lasted for around three years since it was declared a public health emergency of international concern in 2020. Over 635 million confirmed coronavirus disease-2019 (COVID-19) cases, and more than 6.6 million deaths have been reported globally. Besides the direct impact on the health of infected patients, the SARS-CoV-2 pandemic also impacted the clinical outcomes of patients without COVID-19. The incidence of influenza and other notifiable infectious diseases was reduced along with the public health interventions to mitigate the pandemic. However, the impacts of the pandemic and public health intervention on the occurrence of common pediatric infectious diseases remained unknown.

The current study is designed to analyze the trend of the occurrence of pediatric common respiratory and intestinal infectious diseases in southern China before and after the outbreak of the pandemic.

2. Objectives

The current study is designed to explore the impact of the pandemic and public health interventions on the occurrence of pediatric common respiratory and intestinal infectious diseases, so as to provide clinical evidence to support the strategy of public health intervention for children and adolescents.

3. Study design

3.1 General design

A multicentre, retrospective, observational study is designed to explore the trends of pediatric common respiratory and intestinal infectious diseases before and after the pandemic.

3.2 Duration of study

Clinical data will be retrieved from both inpatient and outpatient databases in the participant hospitals for the period from January 1, 2017, to July 31, 2022.

3.3 Participant/ Data selection

Medical data will be included in the current study for the cases that meet the inclusion criteria and do not meet the exclusion criteria.

3.3.1 Inclusion criteria

(a) with one of the below diagnoses: acute upper respiratory tract infection (AURTI), influenza, scarlatina, acute bronchitis, bronchopneumonia, measles, hand, foot, and mouth disease (HFMD), varicella, infectious mononucleosis, rotavirus infection, adenovirus infection, diarrhea, leukemia, and fracture;

(b) aged between 0-18 years.

3.3.2 Exclusion criteria

Aged over 18 years.

4. Data management

4.1 Data handling

Study data will be managed in compliance with local data management requirements for individual sites. Medical records of the subjects will be accessed by the site investigators for data collection. Clinical data will be extracted from the investigational site, including demographic data, annual and monthly cases of pediatric diseases identified by the inclusion criteria.

4.2 Source data and record archiving

Medical data of the subjects will be archived at the investigational site in compliance with local SOP. Study-relative documents will be kept in the study files, and archived in a key-locked filing cabinet at the site. All essential documents will be retained at investigational sites for a minimum of 5 years after the study has finished.

4.3 Data quality

The collected data will be cross-checked by two researchers for quality control, and analyzed by skilled staff with the supervision of a clinical physician and a specialist in epidemiology and hygenic statistics.

5. Statistical methods

A descriptive statistic is employed to analyze the distribution features of variables. Categorical variables are presented as number and frequency rates. Fisher's exact test and χ^2 test are performed using SPSS software (version 26.0, IBM, Armonk, NY, USA) to compare the ratios among groups. Regression analysis is performed using Joinpoint software (version 4.8.0.1) with annual percent change (APC) as a key index to evaluate the trends of annual occurrence of leukemia from 2017 to 2021. Statistical significance is considered if $p < 0.05$. No imputation will be used for missing data.

6. Ethics considerations

6.1 Local regulations/ ICH-GCP

The investigators ensure that the current study is conducted in accordance with the principles of the Declaration of Helsinki, ICH-GCP, and local regulations.

6.2 Informed consent

Informed consent is exempted by the IRB of the leading site since the data are exacted from the medical databases, which are de-identified before being collected for scientific research purposes to protect the privacy of patients.

6.3 Subject confidentiality

The study staff will safeguard the privacy of the subjects' personal data. The personal data will be de-identified before being collected. All documents will be stored securely and only accessible to study staff.

6.4 Independent Ethics Committees

The study protocol and informed consent form will be submitted by the investigators to the EC for written approval. The study procedure could be performed after the EC approval is available.
